# Histology profiling of lung tumors: tru-cut versus full-core system for CT-guided biopsies

**DOI:** 10.1007/s11547-024-01772-4

**Published:** 2024-03-21

**Authors:** Marcello Andrea Tipaldi, Edoardo Ronconi, Nicolò Ubaldi, Fernando Bozzi, Francesco Siciliano, Aleksejs Zolovkins, Gianluigi Orgera, Miltiadis Krokidis, Giulio Quarta Colosso, Michele Rossi

**Affiliations:** 1 Department of Surgical and Medical Sciences and Translational Medicine, “Sapienza” - University of Rome, Rome, Italy; 2 Department of Interventional Radiology, Sant’Andrea University Hospital La Sapienza, Rome, Italy; 3 School of Medicine, National and Kapodistrian University of Athens Areteion Hospital 76, Vas. Sophias Ave, 11528 Athens, Greece

**Keywords:** Lung nodules, Image-guided biopsy, Full-core biopsy, Tru-cut biopsy, CT-guided, Lung neoplasms

## Abstract

**Purpose:**

We aimed to compare the diagnostic yield and procedure-related complications of two different types of systems for percutaneous CT-guided lung biopsy.

**Material and methods:**

All patients with a lung lesion who underwent a CT-guided lung biopsy at our institution, between January 2019 and 2021, were retrospectively analyzed. The inclusion criteria were: (a) Procedures performed using either a fully automated tru-cut or a semi-automated full-core biopsy needle, (b) CT images demonstrating the position of the needles within the lesion, (c) histopathological result of the biopsy and (d) clinical follow-up for at least 12 months and\or surgical histopathological results. A total of 400 biopsy fulfilling the inclusion criteria were selected and enrolled in the study.

**Results:**

Overall technical success was 100% and diagnostic accuracy was 84%. Tru-cut needles showed a significantly higher diagnostic accuracy when compared to full-core needles (91% vs. 77%, *p* = 0.0004) and a lower rate of pneumothorax (31% vs. 41%, *p* = 0.047). Due to the statistically significant different of nodules size between the two groups, we reiterated the statistical analysis splitting our population around the 20 mm cut-off for nodule size. We still observed a significant difference in diagnostic accuracy between tru-cut and full-core needles favoring the former for both smaller and larger lesions (81% vs. 71%, *p* = 0.025; and 92% vs. 81%; *p* = 0.01, respectively).

**Conclusion:**

Our results demonstrated that the use of automated tru-cut needles is associated with higher histopathological diagnostic accuracy compared to semi-automated full-core needles for CTLB.

## Introduction

Lung cancer remains one of the most common causes of cancer death worldwide [1]. The utilization of computed tomography (CT) scans and the implementation of lung cancer screening have resulted in a notable increase in the detection rate of lung lesions. Usually, these lesions necessitate histological confirmation of their pathology before progressing to additional management steps. Tissue diagnosis is of paramount importance in the management of patient with lung cancer not only for histological identification but also to delineate the mutational profile and to deliver targeted treatment [2]. Percutaneous CT-guided lung biopsy (CTLB) represents the primary, minimally invasive, image-guided approach used to obtain tissue diagnosis of lung neoplasms [3, 4]. If feasible, tissue sampling through bronchoscopy would be the first approach; however, the use of a fine needle during bronchoscopy by most operators may provide limited cellular sampling, which may not always be adequate for mutational profiling. Furthermore, most bronchoscopy operators would sample only centrally located lesions, and the procedure requires sedation or even general anesthesia.

CTLB is now a consolidated diagnostic tool, especially for lesions located in the periphery of the lung parenchyma. A couple of small tissue samples are obtained in most cases, offering adequate tissue material for both histological and biomolecular analysis [5]. It also needs to be underlined that negative sampling may occur in some cases due to tissue necrosis or erroneous technique and the method is not free from complications. Several biopsy systems may be used but these are mainly divided into two main categories: the fully automated biopsy needle system and the semi-automated biopsy needle system. Even though both systems have been widely used, there is very limited evidence comparing different biopsy gun systems, making the choice between fully automated and semi-automated systems rather arbitrary [6].

The purpose of our study is to compare two different types of 18G needles systems for CTLB: a fully automated tru-cut (ACECUT 18G-GMG Med Srl) and a semi-automatic full-core needle (BIOMOL 18G-H.S. Spa) in evaluating the histopathological diagnostic yield and the procedure-related complications.

## Material and methods

We conducted a retrospective investigation involving all patients with a lung lesion who underwent a CT-guided biopsy at our institution between January 2019 and 2021. The mentioned period was investigated due to the introduction in the daily practice of a tru-cut needle for lung biopsy, not used before.

The inclusion criteria were: (a) Procedures performed using either one of the following two needle systems: a fully automated tru-cut (ACECUT 18G-GMG Med Srl) or a semi-automatic full core (BIOMOL 18G-H.S. Spa) for new lung lesions, (b) CT images demonstrating the position of the needles within the lesion, (c) histopathological result of the biopsy and (d) clinical follow-up for at least 12 months and\or surgical histopathological results.

All methods or experimental protocols were approved by the local institutional review board and were carried out in accordance with relevant guidelines and were conducted according to the guidelines of the Declaration of Helsinki. Informed consent was obtained from all participants and/or their legal guardians.

### Needle system

The ACECUT needle is a single-use sterilized fully automated biopsy device equipped with a rapid firing side notch. It uses a two-stage biopsy action. ACECUT makes use of a spring action that rapidly propels the inner trocar frontward, with the outer cutting cannula following suit straightaway in a similar forward thrust. As a result, the tissue sample is promptly caught in the side notch of the trocar during the advancement of the cutting cannula. It possesses a function that can individually fire the inner and outer needles with a penetration depth of 22 mm. (There is another available tip of 11 mm not used in our series.) Moreover, it is possible to use a single-use coaxial guide needle with a trocar point, depending on the operators’ preference, to assist in the introduction of the biopsy needle. The lengths available ranges from 75 to 200 mm.

The BIOMOL needle is a single-use sterilized semi-automated biopsy device that necessitates manually advancing the trocar to uncover the side notch. When pressure is applied to its plunger, the cutting cannula is propelled forward, entrapping the specimen in the containing side notch of the trocar. It automates the procedure while keeping sensitivity during the sampling. The size available varies from 16 to 22 gouge with different lengths available.

The size and lengths of each needles are chosen by the operators depending on their preference, the organ and the characteristics of the target lesion.

### Biopsy procedure

All procedures were performed after written informed consent was obtained from the patients.

During the biopsy procedure, preliminary helical CT scan images of the lung were acquired in 1 mm thick slices. From a review of preliminary images, the patient’s position, level of the needle entry site and direction of the approach were planned to provide the most direct route for the biopsy, to go through the shortest track of aerated lung and to avoid bullae and fissures. The procedure was performed by one of four interventional radiologists with more than 5 years of experience on percutaneous CT-guided biopsy or from residents under their direct supervision.

The utilization of the coaxial technique depended on the preference of the operator performing the biopsy. All procedures were performed using a 256 (Brilliance ICT 256, Philips Healthcare, Cleveland, OH, EE, UU) multislice spiral scanner under fluoroscopy guidance.

The radiologist wore a shielding lead apron and was in charge of controlling the CT fluoroscopic exposure using a foot pedal. A sterile field was prepared along the thoracic skin for the proper execution of the procedure. Operators executed the pulmonary biopsy utilizing a real-time CT fluoroscopic method.

The cytopathologist was not present on site during the procedure; hence, biopsy outcome could not be obtained immediately after sampling. Every tissue sampling was conducted under the administration of local anesthesia. Subsequently, thoracic CT scans were acquired to identify potential complications, such as pneumothorax, which may have arisen during the procedure. For patients who exhibited moderate to severe pneumothorax, immediate manual aspiration of air from the pleural space was performed. When the pneumothorax did not reduce, an 8Fr chest drainage tube was placed. Specimens obtained by biopsies were evaluated histologically and cytologically by experienced cytopathologists.

### Investigated variables

Taking into consideration that the population was rather homogeneous in terms of age and sex, the following parameters were assessed: (1) technical success rate, (2) number of samples obtained in a single biopsy procedure, (3) size of the target lesion according to the longest diameter, (4) distance of the target lesion from the pleura access, (5) basal location and (6) rate and type of complications.

Technical success was defined when there was confirmation on the CT fluoroscopic image that the target lesion was touched or completely penetrated by the biopsy needle.

The biopsy sample was considered as “nondiagnostic” if it was not adequate to obtain a specific benign or malignant disease. Histological findings of percutaneous lung biopsy were compared with the final diagnosis obtained after needle biopsy by independent surgical pathology or clinical\radiological follow-up.

Distance of the target lesion from the pleura access was evaluated considering the optimal needle trajectory avoiding big size vessels, visible bronchi and interlobular fissures.

A lesion was categorized as being situated in a lung’s basal segment if, within one CT slice, both a portion of the diaphragm and the neoplasm were present, a feature that has been noted as a limiting factor of percutaneous lung biopsies [7].

As complications, the incidence of pneumothorax, hemothorax and hemoptysis was evaluated. Regarding pneumothorax, the rates of administration of manual aspiration and tube placement were investigated.

All the dependent variables were compared between the ACECUT group and the BIOMOL group.

### Statistical analysis

Continuous variables were reported as mean (standard deviation, SD) or median (interquartile range, Q1–Q3). Distribution was checked for normality using the Shapiro–Wilk test. Categorical variables were presented as absolute number (*n*) and relative percentage frequency (%).

Variables were tested for significant differences between the two groups of full-core needle versus tru-cut needles.

To compare continuous variables between two groups, a two-tailed T-test was used when normally distributed, whereas a Mann–Whitney U test was used when not normally distributed. Fisher exact test was performed to test the association between categories for dichotomous variables.

Post hoc power analysis has been conducted with the software G*Power version 3.1.

A multivariable analysis was performed running a binary logistic regression with stepwise forward selection, to further investigate differences in the two population.

A *p* value of 0.05 was considered statistically significant.

The software BM SPSS Statistics for Windows, Version 24.0 was used for all the statistical analyses.

## Results

**Fig. 1:**
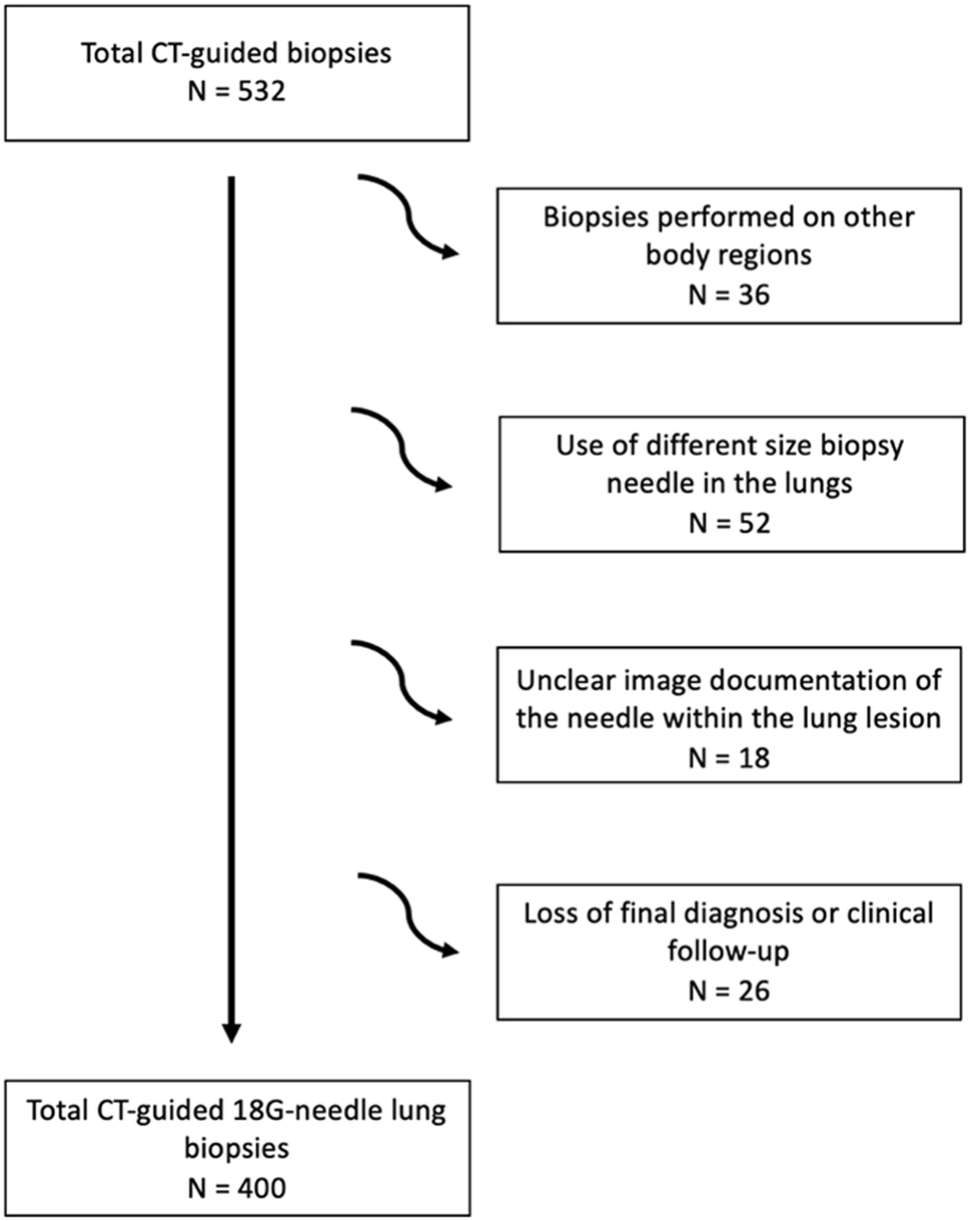
Flowchart of the study population and reasons for exclusion

Between January 2019 and 2021, a total of 532 CT biopsies were performed of which 400, fulfilling the inclusion criteria, were enrolled in the study. A total of 132 patients were excluded due to their failure to meet the inclusion criteria stated in the article, including procedures on other body regions (such us bone or soft paracostal tissue), the use of different biopsy guns from the ones stated in the study design, CT images that did not clearly display the correct needle positioning within the lung nodule, contaminated tissue specimens from external factors and lack of final diagnosis by independent surgical pathological findings or clinical follow-up at 12 months (Fig. 1).

Overall technical success was 100% and diagnostic accuracy was 84% (adequate samples). Pneumothorax occurred in 142 (35%) patients but only 10 (4%) required chest tube placement. 23% of small alveolar hemorrhage occurred not requiring any treatment.

Regarding the type of needle used, two groups with equal number of patients were identified: the tru-cut needles with 200 patients and the full core with other 200 patients. Tru-cut needles showed a significantly higher diagnostic accuracy when compared to full-core needles (91% vs. 77%, respectively, *p* = 0.0004, power 87%) and a lower rate of pneumothorax (31% vs. 41%, respectively, *p* = 0.047). The two groups showed no significant difference in terms of age, sex, nodule location, number of samples obtained and pleura to nodule distance but differed significantly in nodule size (33 ± 18 mm vs. 31 ± 21 mm, *p* = 0.019) as shown in Table 1.

**Table 1:** Patient demographics and characteristics

	All	Tru-cut (*n* 200)	Full core (*n* 200)	*P* value
Age ± SD, years	69 ± 11	69 ± 12	70 ± 10	0.934
Sex, male	247(62%)	125(63%)	122(61%)	0.836
Basal location	104(26%)	61(31%)	43(22%)	0.052
Pleura to nodule distance	10 IQR 0–29 mm	7 IQR 0–25 mm	13 IQR 0–29 mm	0.088
Nodule size	32 ± 19 mm	33 ± 18 mm	31 ± 21 mm	0.019
Pneumothorax rate	142(35%)	31%	41%	0.047

Due to the statistically significant different of nodules size between the two groups, we conducted a subanalysis splitting our population around the 20 mm cut-off for nodule size. A total of 259 patients underwent a biopsy for a lesion larger than 2 cm (Group 1, Table 2) and 141 for a lesion smaller than 2 cm (Group 2, Table 3). In Group 1 the tru-cut system was used in 138 patients (Group 1a) and the full-core system in 121 (Group 1b). In Group 2 the tru-cut system was used in 62 (Group 2a) and the full core in 79 (Group 2b) patients.

**Table 2:** Group 1: Patient demographics and characteristics for large lesions > 20 mm

	Tru-cut (1a) (*n* 138)	Full core (1b) (*n* 121)	*P* value
Age ± SD, years	69 ± 12	69 ± 11	0.904
Sex, male	89(64)	80(66)	0.795
Basal location	29(21)	23(19)	0.3
Pleura to nodule distance	0 IQR 0–20 mm	10 IQR 0–28 mm	0.096
Nodule size	41 ± 17	41 ± 21	0.32
Pneumothorax rate	30%	42%	0.053

**Table 3:** Group 2: Patient demographics and characteristics for small lesions < 20 mm

	Tru-cut (2a) (*n* 62)	Full core (2b) (*n* 79)	*P* value
Age ± SD, years	68 ± 12	71 ± 10	0.381
Sex, male	33(53)	45(57)	0.734
Basal location	12(19)	20(25)	0.426
Pleura to nodule distance	14 IQR 0–31 mm	14 IQR 0–29 mm	0.802
Nodule size	16 ± 3	15 ± 4	0.083
Pneumothorax rate	31%	38%	0.38

We reiterated the statistical analysis in the two groups, observing a significant difference in diagnostic accuracy between tru-cut and full-core needles favoring the former for both smaller (Group 2a–b) and larger lesions (Group 1a–b) (81% vs. 71%, *p* = 0.025; and 92% vs. 81%; *p* = 0.01, respectively), as shown in Table 4. The multivariate analysis confirmed no statistical differences between Group 1a and b and Group 2a and b regarding pneumothorax rate, age, sex, nodule location, pleura to nodule distance and dimension, as shown in Tables 2 and 3.

**Table 4:** Biopsy diagnostic accuracy

	Tru-cut (%)	Full core (%)	*P* value
All (*n* 400)	91	77	0.0004
Small lesions (< 20 mm) (n 141)	81	71	0.025
Large lesions (> 20 mm) (*n* 259)	92	81	0.01

**Fig. 2:**
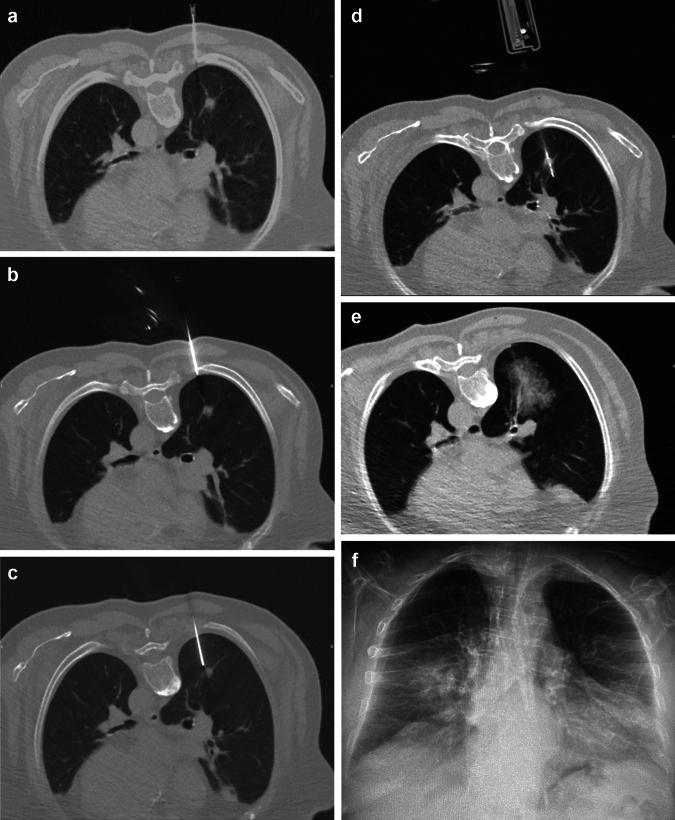
**a** Local anesthesia performed from the subcutaneous layers to the extra-pleural space. **b** Coaxial needle is positioned outside the pleura prior to advancing it as close to the nodule as possible. **c** Coaxial needle pierces the lung in one step and is positioned in the vicinity of the nodule. **d** The automatic ACECUT biopsy gun is inserted through the coaxial needle and is fired for material retrieval. **e** CT scan after the biopsy documents hematic alveolar hemorrhage due to the central position of the nodule in communication with larger pulmonary vessels. No pneumothorax is documented. Patient spontaneously resolved hemoptysis 1 h after the procedure. **f** X-ray at 3 h post the procedure confirms the absence of pneumothorax with medio-basal opacity consistent with alveolar hemorrhage. No pleural effusion is seen.

Case 1: Sequential steps of the percutaneous transthoracic biopsy procedure, utilizing ACECUT 18G needle, in a patient with a suspicious pulmonary nodule (1 cm), located centrally in the apical segment of the right inferior pulmonary lobe (Fig. 2)

.

**Fig. 3:**
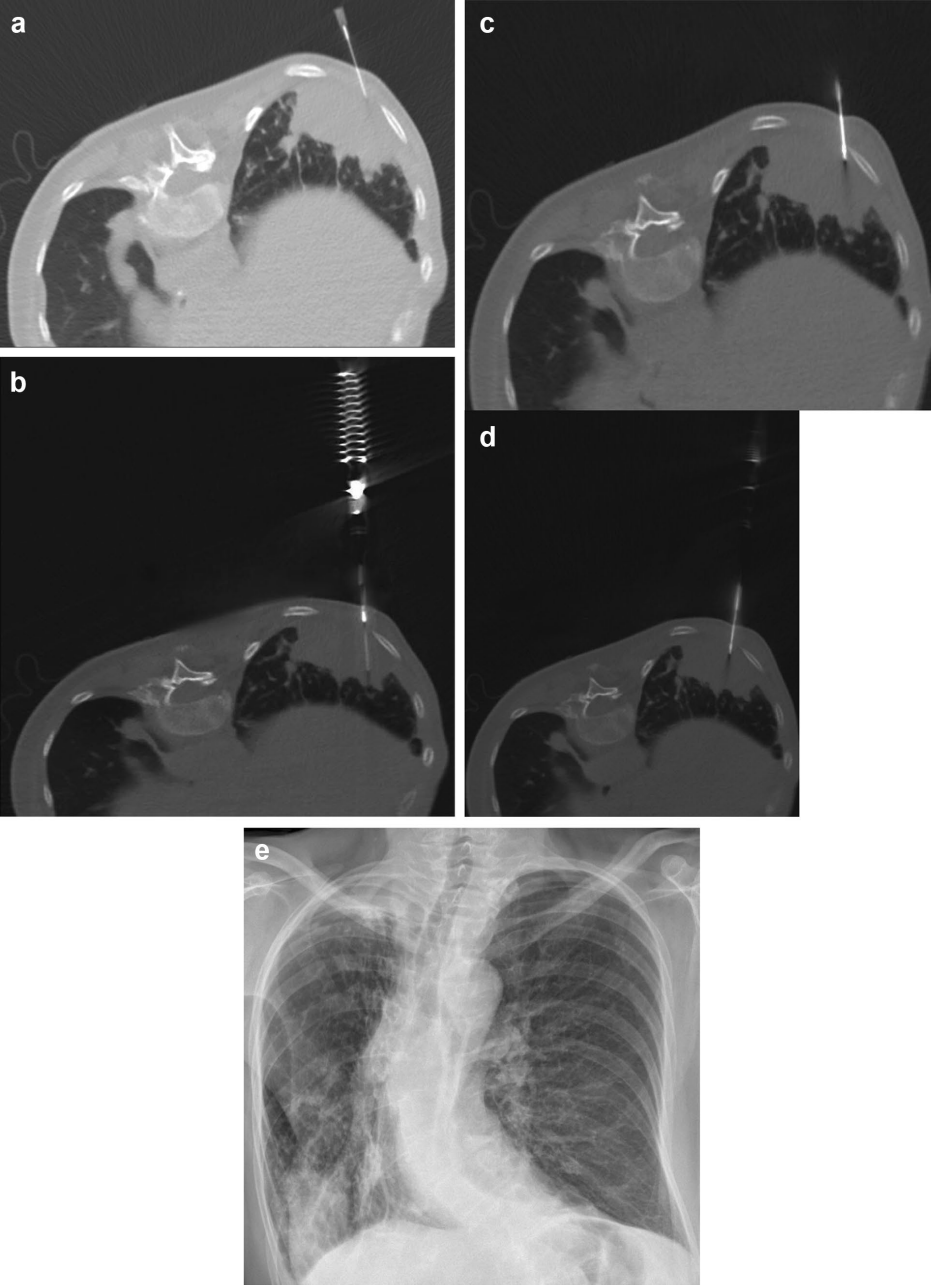
**a** Local anesthesia from the subcutaneous layers to the extra-pleural space. **b** The trocar is manually advanced to uncover the side notch. **c**–**d** When pressure is applied to its plunger, the cutting cannula is propelled forward, entrapping the specimen in the containing side notch of the trocar. The trocar is advanced, rotated and retracted several times at multiple angles to entrap the most material possible. **e** RX 3 h post the procedure confirms the absence of neither pneumothorax nor alveolar hemorrhage

Case 2: Sequential steps of the percutaneous transthoracic biopsy procedure, utilizing BIOMOL 18G needle, in a patient with a suspicious pulmonary mass (> 3 cm), located in posterior basal segment of the left inferior pulmonary lobe (Fig. 3).

## Discussion

In the overall study population, the fully automated tru-cut needles were associated to significantly higher diagnostic histopathological accuracy compared to semi-automated full-core needles, respectively, of 91% and 77% (*p* = 0.0004), and a lower rate of pneumothorax, respectively, of 31% and 41% (*p* = 0.047).

New lung lesions visualized on chest imaging should undergo accurate multidisciplinary assessment in order to guide patients through the correct management workflow [8]. Lung biopsy is necessary for a specific diagnosis and in view of a treatment plan. Various techniques are available to obtain lung tissue for histological exam, including percutaneous transthoracic, transbronchial, video-assisted thoracic surgery and open biopsy [9]. Indications for biopsy in lesions with a non-negligible risk of pathology, according to the BTS guidelines, include: persistent or new enlarging focal lesions not amenable for diagnosis by either CT alone, sputum, serology or bronchoscopy [10]. Starting from 1976 with the first report of CT-guided lung biopsy, this tool has been widely used as a valid diagnostic method for suspicious pulmonary lesions. With the advent of increased diagnosis of small lesions owing to improved screening organization, new perspectives regarding diagnosis arose. Percutaneous biopsy can be carried out as core biopsy or fine-needle aspiration biopsy, the prior allowing for more numerous and larger tissue specimens retrieval, best for tissue characterization thus leading to higher definite diagnosis [11], especially for smaller lesions [11]. In fact, automated lung biopsy with or without coaxial system has been proposed as first-line diagnostic tool for small and larger (< 2 cm >) lung nodules, owing to a good diagnostic accuracy [12, 13], however at the cost of higher complications [14, 15].

In our institution, we adopt automatic and semi-automatic biopsy guns with or without coaxial technique. This retrospective study enrolled a total of 400 lung nodules biopsied using either automatic tru-cut-type biopsy gun (ACECUT 18G-GMG Med Srl) or semi-automatic full-core-type biopsy gun (BIOMOL 18G-H.S. Spa). To the best of our knowledge, only one other study has compared automatic and semi-automatic needles for CT-guided lung biopsy in the diagnostic accuracy and overall complications [6], reporting better results in favor of automatic guns. Outcomes that were also reflected in biopsies performed on other organs [16, 17]. We hereby confirm that these techniques are efficacious and highly accurate, with relatively low complications associated. In fact, we report a high (84%) overall diagnostic accuracy of CT-guided percutaneous pulmonary biopsy in patients with yet unidentified lung lesions, in accordance with previous studies [18].

Specifically, our research paper demonstrates that the automated tru-cut-type biopsy gun showed significantly higher diagnostic accuracy than the semi-automated full-core-type biopsy gun, in both small and large lesions (81% vs. 71% *p* = 0.025 and 92% vs. 81% *p* = 0.01, respectively). Only one other study compared automated with semi-automated needles for lung tumor biopsies, however in a much smaller population [6]. They obtained similar results to ours, showing a greater frequency of sufficient tissue sampling (100% vs. 83%) and higher diagnostic accuracy (97.6% vs. 83.3%) [6]. As already documented in the literature [13], we confirm that nodule size was a decisive factor in diagnostic accuracy. Especially true when considering small lesions that were associated with lower diagnostic accuracy and can be difficult to reach, interventional radiologists should take advantage of the best available instruments at hand to improve diagnostic results. Furthermore, the automated nature of the tru-cut gun reduces the risk of operator-dependent errors, such as suboptimal nodule sampling, especially true in small lesions, and allows for faster sampling and operator convenience which in return may enhance overall success rates. Moreover, in large lesions we suggest using coaxial technique allowing the withdrawal of an increased number and volume of tissue samples, thus improving the diagnostic accuracy. Nonetheless, large lesions were more susceptible to misdiagnosis [19].

Overall, our experience routes in favor of using automatic tru-cut needles independently of lung nodule size, also to minimize operator-dependent error associated with the semi-automatic needle. The fully automatic system operates with a mechanism that ensures consistent and precise tissue sampling, facilitating the retrieval of larger and more representative specimens that may improve the accuracy of the histopathological diagnosis. It is always recommended to consider multiple factors when selecting the appropriate biopsy system for CT-guided lung biopsies, including operator experience and individual patient characteristics. One aspect to consider is the assessment of the standardized uptake value from a PET exam whenever possible, shortly prior to the biopsy and ideally with the assistance of a software that superimposes the images, as the value of 18F-FDG uptake is associated to reduce false negative results and inadequate samples [20–22].

In the literature, the most common complication associated with percutaneous lung biopsy is pneumothorax, ranging between 20 and 27%, with roughly 7–14% requiring interventional treatment [15, 23–26].

In this study all adverse events were considered to be minor. Automatic biopsy with tru-cut needles was associated with lower incidence of pneumothorax, compared to full-core needles, especially in larger lesions (30% vs. 42%), in accordance with Yoshimatsu et al. [6]. Intuitively, semi-automatic needles are more traumatic than fully automatic guns, as they require more manual advancement of the trocar to allow the necessary core drilling. Other complications include intraparenchymal bleeding with an incidence of 11–12% [6, 27, 28]. In our series, hemoptysis due to small alveolar hemorrhage occurred in 23% of the procedure even if it did not require any intervention, Grade 1 of the CIRSE complication.

This study has several limitations. This is a single-center non-randomized study and selection bias was present. The choice of using either tru-cut or full-core needle and the number of tissue sample retrieval did not follow any criteria. Also, deciding whether to use coaxial technique for the automatic needle did not follow any criteria. Future studies could be done to standardize these criteria in order deepen our results favoring fully automatic needles rather than semi-automatic needles.

## Conclusions

In conclusion, percutaneous CTLB is an accurate and tolerably safe method to diagnose new suspicious pulmonary nodules. This study is one of few that compares the two most common techniques involved in the mini-invasive diagnostic methods. Clearly, the use of automated tru-cut needles, compared to semi-automated full-core needles, is associated with higher diagnostic accuracy. These results have important implications for clinicians and highlight the significance of selecting the appropriate needle system for CT-guided lung biopsies to improve diagnostic outcomes.

## References

[1] Bade BC, Dela Cruz CS (2020). Lung Cancer 2020: epidemiology, etiology, and prevention. Clin Chest Med.

[2] Mayekar MK, Bivona TG (2017). Current landscape of targeted therapy in lung cancer. Clin Pharmacol Ther.

[3] MacMahon H, Naidich DP, Goo JM (2017). Guidelines for management of incidental pulmonary nodules detected on CT images: from the Fleischner society 2017. Radiology.

[4] Aberle DR, Adams AM, National Lung Screening Trial Research Team (2011). Reduced lung-cancer mortality with low-dose computed tomographic screening. N Engl J Med.

[5] Duka E, Ierardi AM, Floridi C, Terrana A, Fontana F, Carrafiello G (2017). The role of interventional oncology in the management of lung cancer. Cardiovasc Intervent Radiol.

[6] Yoshimatsu R, Yamagami T, Tanaka O (2012). Comparison of fully automated and semi-automated biopsy needles for lung biopsy under CT fluoroscopic guidance. Br J Radiol.

[7] Tipaldi MA, Ronconi E, Krokidis ME (2022). Diagnostic yield of CT-guided lung biopsies: how can we limit negative sampling?. Br J Radiol.

[8] Manhire A, Charig M, Clelland C (2003). Guidelines for radiologically guided lung biopsy. Thorax.

[9] Modi P, Uppe A (2023) Lung biopsy techniques and clinical significance. In: StatPearls. StatPearls Publishing. Accessed from 10 Jul 2023. http://www.ncbi.nlm.nih.gov/books/NBK563153/33085300

[10] Baldwin DR, Callister MEJ, Graham R, Gleeson F, Members of the Guideline Development Grou (2016). Pulmonary nodules again? The 2015 British thoracic society guidelines on the investigation and management of pulmonary nodules. Clin Radiol.

[11] Choi SH, Chae EJ, Kim JE (2013). Percutaneous CT-guided aspiration and core biopsy of pulmonary nodules smaller than 1 cm: analysis of outcomes of 305 procedures from a tertiary referral center. AJR Am J Roentgenol.

[12] Yildirim E, Kirbas I, Harman A (2009). CT-guided cutting needle lung biopsy using modified coaxial technique: factors effecting risk of complications. Eur J Radiol.

[13] Huang MD, Weng HH, Hsu SL (2019). Accuracy and complications of CT-guided pulmonary core biopsy in small nodules: a single-center experience. Cancer Imaging.

[14] Beslic S, Zukic F, Milisic S (2012). Percutaneous transthoracic CT guided biopsies of lung lesions; fine needle aspiration biopsy versus core biopsy. Radiol Oncol.

[15] Winokur RS, Pua BB, Sullivan BW, Madoff DC (2013). Percutaneous lung biopsy: technique, efficacy, and complications. Semin Intervent Radiol.

[16] Sridharan R, Yunos SM, Aziz S (2015). Comparison on the use of semi-automated and automated core biopsy needle in ultrasound guided breast biopsy. Med J Malays.

[17] Park JY, Yi SY, Baek SH, Lee YH, Kwon HJ, Park HJ (2022). Diagnostic efficacy, performance and safety of side-cut core needle biopsy for thyroid nodules: comparison of automated and semi-automated biopsy needles. Endocrine.

[18] Zhang HF, Zeng XT, Xing F, Fan N, Liao MY (2016). The diagnostic accuracy of CT-guided percutaneous core needle biopsy and fine needle aspiration in pulmonary lesions: a meta-analysis. Clin Radiol.

[19] Yeow KM, Tsay PK, Cheung YC, Lui KW, Pan KT, Chou ASB (2003). Factors affecting diagnostic accuracy of CT guided coaxial cutting needle lung biopsy: retrospective analysis of 631 procedures. J Vasc Interv Radiol.

[20] Piacentino F, Fontana F, Zorzetto G (2023). Could maximum SUV be used as imaging guidance in large lung lesions biopsies? Double sampling under PET-CT/Xper guide fusion imaging in inhomogeneous lung uptaking lesions to show that it can make a difference. Technol Cancer Res Treat.

[21] Fontana F, Piacentino F, Ierardi AM (2021). Comparison between CBCT and Fusion PET/CT-CBCT guidance for lung biopsies. Cardiovasc Intervent Radiol.

[22] Caruso D, Zerunian M, Daffina J (2021). Radiomics and functional imaging in lung cancer: the importance of radiological heterogeneity beyond FDG PET/CT and lung biopsy. Eur J Radiol.

[23] Covey AM, Gandhi R, Brody LA, Getrajdman G, Thaler HT, Brown KT (2004). Factors associated with pneumothorax and pneumothorax requiring treatment after percutaneous lung biopsy in 443 consecutive patients. J Vasc Interv Radiol.

[24] Yeow KM, Su IH, Pan KT (2004). Risk factors of pneumothorax and bleeding: multivariate analysis of 660 CT-guided coaxial cutting needle lung biopsies. Chest.

[25] Saji H, Nakamura H, Tsuchida T (2002). The incidence and the risk of pneumothorax and chest tube placement after percutaneous CT-guided lung biopsy: the angle of the needle trajectory is a novel predictor. Chest.

[26] Huo YR, Chan MV, Habib AR, Lui I, Ridley L (2020). Pneumothorax rates in CT-Guided lung biopsies: a comprehensive systematic review and meta-analysis of risk factors. Br J Radiol.

[27] Li Y, Du Y, Yang HF, Yu JH, Xu XX (2013). CT-guided percutaneous core needle biopsy for small (≤20 mm) pulmonary lesions. Clin Radiol.

[28] Yang W, Sun W, Li Q (2015). Diagnostic accuracy of CT-guided transthoracic needle biopsy for solitary pulmonary nodules. PLoS ONE.

